# GC-MS Characterization of Antibacterial, Antioxidant, and Antitrypanosomal Activity of *Syzygium aromaticum* Essential Oil and Eugenol

**DOI:** 10.1155/2021/6663255

**Published:** 2021-02-20

**Authors:** Amanda Mara Teles, João Victor Silva-Silva, Juan Matheus Pereira Fernandes, Ana Lucia Abreu-Silva, Kátia da Silva Calabrese, Nestor Everton Mendes Filho, Adenilde Nascimento Mouchrek, Fernando Almeida-Souza

**Affiliations:** ^1^Pós-Graduação em Saúde do Adulto, Universidade Federal do Maranhão, São Luís 65065-545, Brazil; ^2^Laboratório de Imunomodulação e Protozoologia, Instituto Oswaldo Cruz, Fiocruz, Rio de Janeiro 21040-900, Brazil; ^3^Pós-Graduação em Ciência Animal, Universidade Estadual do Maranhão, São Luís 65055-310, Brazil; ^4^Laboratório de Controle de Qualidade de Alimentos e Água, Universidade Federal do Maranhão, São Luís 65065-545, Brazil

## Abstract

*Syzygium aromaticum* has a diversity of biological activities due to the chemical compounds found in its plant products such as total phenolic compounds and flavonoids. The present work describes the chemical analysis and antimicrobial, antioxidant, and antitrypanosomal activity of the essential oil of *S. aromaticum*. Eugenol (53.23%) as the major compound was verified by gas chromatography-mass spectrometry. *S. aromaticum* essential oil was more effective against *S. aureus* (MIC 50 *μ*g/mL) than eugenol (MIC 250 *μ*g/mL). Eugenol presented higher antioxidant activity than *S. aromaticum* essential oil, with an EC_50_ of 12.66 and 78.98 *µ*g/mL, respectively. *S. aromaticum* essential oil and eugenol exhibited *Trypanosoma cruzi* inhibitory activity, with IC_50_ of 28.68 ± 1.073 and 31.97 ± 1.061 *μ*g/mL against epimastigotes and IC_50_ of 64.51 ± 1.658 and 45.73 ± 1.252 *μ*g/mL against intracellular amastigotes, respectively. Both compounds presented low cytotoxicity, with *S. aromaticum* essential oil displaying 15.5-fold greater selectivity for the parasite than the cells. Nitrite levels in *T. cruzi*-stimulated cells were reduced by essential oil (47.01%; *p* = 0.002) and eugenol (48.05%; *p* = 0.003) treatment. The trypanocidal activity of *S. aromaticum* essential oil showed that it is reasonable to use it in future research in the search for new therapeutic alternatives for trypanosomiasis.

## 1. Introduction

The clove (*Syzygium aromaticum*) belongs to the family Myrtaceae originating from the Maluku Islands, in eastern Indonesia. It is a tree up to 12 meters high that can be grown in coastal areas at high altitudes, and its best known part is the flower buds that are produced after four years of planting and collected before flowering [1, 2]. The main chemotype of flower buds essential oil is its major compound, eugenol, a volatile phenylpropanoid widely used in the pharmaceutical industry. It also contains eugenol acetate, *β*-caryophyllene, and humulene [3]. *S. aromaticum* has demonstrated antiviral activity against food-borne viruses [4], antimicrobial activity, with low concentrations able to inhibit bacteria growth [5], and effectiveness in the treatment of bacterial infections [6].

Infectious diseases can be caused by several species, with the main pathogens related to bacteria. These infections are a serious problem in public health, due to their direct impact on society. They are caused by pathogenic microorganisms that invade the host organism, overcome its defenses, and cause tissue damage [7]. A further concern is the high rates of resistance of microorganisms, which occur in diseases such as food poisoning, one of the most common causes of death in developing countries. Most reports are associated with bacterial contamination, which requires the development of new antimicrobial agents with the ability to interfere in various activities of the bacterial cell [8].

Other diseases that have attracted attention due to their impact on public health are those caused by protozoa, such as Chagas disease, caused by the protozoan *Trypanosoma cruzi*. *T. cruzi* is a parasite with continental dispersion present in 21 countries in Latin America. There is a worldwide estimate of around 6 to 7 million infections and approximately 75 million living in endemic areas at risk of infection [9]. Nifurtimox and benznidazole are the only treatments used in patients with Chagas disease, causing about 40% of side effects in adult patients, making treatment adherence difficult [9–11]. There are still cases of strains resistant to these treatments [12].

Natural products have a chemical composition rich in secondary metabolisms, which play an important role in plant physiology. They have a variety of potential biological benefits, such as antioxidant, anti-inflammatory, anticancer, antibacterial, and antifungal activities, which act as its defense mechanism against predatory microorganisms. In addition, there are currently hundreds of drugs based on active compounds isolated from plants [13]. Products of plant origin have been described throughout history as therapeutic resources and used in several nations, with ancient civilizations using plant food and other substances as medicine. Their experiments with herbs resulted in both successes and failures, with the latter being associated with serious adverse effects. The discovery of the useful and harmful properties of plants helps in their use [14]. Plants are no longer merely a therapeutic option for needy populations; they have become a promising source of new molecules of therapeutic interest. Therefore, many species still need to be investigated.

In view of the aforementioned research, the use of natural origin products as antimicrobials was evident. The exploration of the biological activity of chemical substances in medicinal plants constitutes a potential form of alternative disease control, generating the development of models that make the sustainable production systems and the conservation of biodiversity and natural resources feasible. Thus, this research aimed to study the chemical properties of the essential oil of *S. aromaticum*, as well as verifying its possible antimicrobial, antioxidant, and antitrypanosomal properties.

## 2. Materials and Methods

### 2.1. Reagents

Anhydrous sodium sulfate, ethanol, ethyl acetate, dimethyl sulfoxide (DMSO), eugenol, 2,2′-azino-bis (3-ethylbenzothiazoline-6-sulfonic acid) diammonium salt (ABTS), penicillin, streptomycin, N-benzyl-2-nitro-1H-imidazole-1-acetamide (benznidazole), Brewer thioglycollate medium, RPMI 1640 medium, 3-(4,5-dimethyl-2-thiazolyl)-2, 5-diphenyl-2H-tetrazolium bromide (MTT), sulfanilamide, H_3_PO_4_, N-(1-naphthyl)ethylenediamine, and sodium nitrite were purchased from Sigma, St Louis, MO, USA. Giemsa's azur-eosin-methylene blue, Brain Heart Infusion broth, Mueller–Hinton agar, and Mueller–Hinton broth were purchased from MERK, Darmstadt, Germany. Fetal bovine serum (FBS) was purchased from Gibco, Gaithersburg, MD, USA.

### 2.2. Plant Material

The floral buds of *S. aromaticum* were purchased from the central market of the city of São Luís, Maranhão, Brazil (2° 31′ 48″ S, 44° 18′ 10″ W). For the extraction of oil, dry flower buds were selected in an oven with air circulation at 37 °C/48 h, and soon afterwards they were sprayed in a knife mill in the Physics-Chemistry Laboratory of the Food and Water Quality Control Program (PCQA), Federal University of Maranhão.

### 2.3. Essential Oil Extraction

The extraction of the essential oil of *S. aromaticum* was carried out with 300 g of the ground vegetable product, and it was diluted in water in the proportion of 1 : 10 by hydrodistillation using the Clevenger system for 3h at 100 °C. The essential oil collected was dried with anhydrous sodium sulfate (Na_2_SO_4_), and the final volume found was used to determine the yield through the mass/volume ratio by measuring the density. Mass/volume ratios were calculated from the mass (g) of the initial vegetal material and the volume (mL) of essential oil obtained after extraction. The essential oil samples were kept at 25°C and then weighed. For the verification of biological activity *in vitro*, the essential oil and the reference drugs were diluted in DMSO, and subsequently serial dilutions were made in an appropriate culture medium until reaching a final concentration below 1% DMSO.

### 2.4. Physical-Chemical Analysis of Essential Oil

Some physical-chemical analyses were performed on the essential oil of *S. aromaticum* for density measured with a glass pycnometer, refractive index calculated with ABBE 2WAJ refractometer (PCE Instruments, Southampton, United Kingdom), color and appearance that were visually verified by three different people, and determination of solubility that is carried out through the ratio of 1: 1 of oil and ethanol 80% until its complete solubilization.

### 2.5. Gas Chromatography-Mass Spectrometry (GC-MS)

The chemical characterization of the essential oil of the floral buds of *S. aromaticum* was determined by gas chromatograph coupled to Shimadzu QP5000 mass spectrometer (Shimadzu, Kyoto, Japan), equipped with a capillary column ZB-5ms (5% phenyl arylene) 95% dimethylpolysiloxane, with HP 5MS electronic impact detector of 70 eV (40-500 Da) and transfer temperature of 280°C. Aliquots were injected in splitless mode with a volume of 0.3 µL in ethyl acetate (automatic injector CP-8410), fixing the following conditions: high purity helium as carrier gas; injector temperature maintained at 280°C, split mode (1 : 10); followed by an initial temperature of 40°C and a final temperature of 300°C, an initial time of 5 min and a final time of 7.5 min at 8/min.

### 2.6. Antioxidant Assay

The determination of antioxidant activity was carried out according to the methodology suggested by Re et al., with modifications [15]. The test was carried out with a radical of ABTS which was prepared by the reaction of 5.0 mL of a 3.840 *μ*g/mL solution of ABTS with 88 *µ*L of the 37.840 *μ*g/mL potassium persulfate solution. The mixture was left in the dark at room temperature for 16 hours. Immediately after mixing, it was diluted in ethanol until an absorbance of 0.7 at 734 nm was obtained. In a dark environment, an aliquot of 30 *µ*L of each concentration of essential oil (200 to 15 *μ*g/mL) and eugenol (90 to 5 *μ*g/mL) was transferred in test tubes containing 3.0 mL of the radical cation ABTS and homogenized in a tube shaker, and after 6 minutes the absorbance of the reaction mixture was read on a spectrophotometer at a length of 734 nm. The tests were performed in triplicate, and the capture of the free radical was expressed as a percentage of inhibition (% I) of the ABTS radical cation according to the following equation: % inhibition = (absorbance of the solution of the ABTS radical - absorbance of the sample)/(ABTS absorbance radical solution) × 100 [16]. It was also determined that the efficient concentration or EC50% represents the concentration of the sample necessary to sequester 50% of the ABTS root, in which the essential oil will be considered active when it has EC50% < 500 *μ*g/mL [17].

### 2.7. Bacterial Strains and Culture Conditions

The tests were carried out at the Microbiology Laboratory of Food and Water Quality Control at the Federal University of Maranhão (PCQA-UFMA). Microbial strains from the American Type Culture Collection (ATCC), *Escherichia coli* (ATCC 25922), *Staphylococcus aureus* (ATCC 12600), and *Pseudomonas aeruginosa* (ATCC 27853), were tested at a cellular concentration of 10^8^ UFC/mL following the McFarland scale, recommended by the Clinical and Laboratory Standards Institute [18].

### 2.8. Antimicrobial Assays

The disk diffusion test was performed using the 75 *μ*L inoculum of the microbial suspension (1.5 × 10^8^ CFU/mL) seeded on Mueller–Hinton agar. Soon after, the disks containing 50 *µ*L of essential oil and eugenol were fixed on the agar surface. The plates were incubated in a bacteriological oven at 35°C for 24 hours. The diameters of the inhibition halos were measured, including the disk diameter, in triplicate [19]. After checking the antimicrobial potential on the strains tested by the disk diffusion method, the oil was subjected to the determination of the minimum inhibitory concentration (MIC), in which an aliquot of the essential oil of *S. aromaticum* and eugenol was transferred to a test tube containing Mueller–Hinton broth. Then, serial dilutions were performed, resulting in concentrations of 1000, 500, 250, 200, 100, 75, 50, 25, 15, and 5 *μ*g/mL. Microbial suspensions containing 1.5 × 10^8^ CFU/mL were added at each concentration and incubated at 35°C for 24 h. After the incubation period, the MIC of the oil was determined, being defined as the lowest concentration that visibly inhibited bacterial growth (absence of visible turbidity) [18].

### 2.9. Parasites

Trypomastigote forms of *Trypanosoma cruzi* (SC2005 strain) were obtained from Vero cells infected and used to infect the macrophages. Epimastigote forms originated from the suspension of cell culture trypomastigotes in 3 mL of LIT medium supplemented with 10% fetal bovine serum (FBS), 100 U/mL of penicillin, and 100 *μ*g/mL of streptomycin and were incubated in an oven at 28°C until complete differentiation of parasites.

### 2.10. Antiepimastigote Assay

Epimastigote forms of *T. cruzi* (10^6^ parasites/mL) from a 2–4-day-old culture were added to 96-well plates, followed by the addition of different concentrations of either *S. aromaticum* essential oil or eugenol (1000–7.81 µg/mL), obtained by serial dilutions (1 : 2), at a final volume of 100 *µ*L per well, for 72 hours. The controls were identified as follows: blank (wells without parasites), untreated control (parasites and DMSO 1%), and reference drug (benznidazole). With the aid of the hemocytometer and light microscopy, viability was evaluated by counting parasites, and the results were used to calculate the IC_50_ (50% inhibition of parasite growth) following the formula: IC_50_ = (sample counting)/(control counting) × 100 [20].

### 2.11. Animals and Ethical Statements

Animals were purchased from the Institute of Science and Technology in Biomodels of the Oswald Cruz Foundation. All procedures involving female 4–6-week-old BALB/c mice were performed in accordance with the National Council for the Control of Animal Experimentation (CONCEA) and approved by the Ethics Committee on Animal Care and Utilization (CEUA/IOC-L018/2018).

### 2.12. Obtaining Peritoneal Macrophages and Cell Culture

Peritoneal macrophages were obtained from BALB/c mice, elicited for 72 hours with 3 mL 3% Brewer thioglycollate medium, and maintained in RPMI 1640 medium supplemented with 10% FBS, 100 U/mL of penicillin, and 100 *µ*g/mL of streptomycin, overnight at 37°C and 5% CO_2_.

### 2.13. Cytotoxicity Assay

Different concentrations, obtained by serial dilutions (1 : 2), of essential oils (1000–7.8 *μ*g/mL) or benznidazole (200–0.78 *μ*g/mL) up to a final volume of 100 *μ*L per well were placed in 96-well plates with peritoneal macrophages (5 × 10^5^ cells/mL). The controls were categorized as blank (wells with culture medium without cells), untreated control (cells and DMSO 1%), and reference drug (benznidazole). After 24 h, the cell viability was analyzed by the MTT colorimetric method [21]. Absorbance was measured in a spectrophotometer at 570 nm wavelength. The concentration inhibiting 50% of cell growth (CC_50_) was calculated following the formula: CC_50_ = (sample absorbance-blank absorbance)/(control absorbance-blank absorbance) × 100 [22].

### 2.14. Activity against Intracellular Amastigotes and Selectivity Index (SI)

Initially, trypomastigote forms of *T. cruzi* from cultured Vero cells were obtained *in vitro*. BALB/c peritoneal macrophages, cultured in 24-well plates (5 × 10^5^ cells/well) with coverslips, were infected with trypomastigotes using the ratio of parasite/cell 10 : 1, at 37°C and 5% CO_2_ for 6 h. Afterwards, well plates were washed with phosphate-buffered saline (PBS, pH 7.2) to remove the noninternalized parasites. The infected cells were treated with different concentrations of *S. aromaticum* essential oil or eugenol (200–12.5 *µ*g/mL) for 24 h. For light microscopy analysis, the coverslips with the infected and treated cells were fixed with Bouin and stained with Giemsa's azur-eosin-methylene blue. The IC_50_ was calculated from the total of intracellular amastigotes from 100 cells. The mean number of amastigotes per cell was obtained from the number of intracellular amastigotes in 100 cells divided by the number of infected cells. Benznidazole was used as the reference drug. The selectivity index (SI) was obtained from the ratio between cytotoxicity of BALB/c peritoneal macrophages and activity against intracellular amastigote (SI = CC_50_/IC_50_) [23].

### 2.15. Nitrite Quantification

BALB/c peritoneal macrophages (5 × 10^6^ cells/mL) were treated with *S. aromaticum* essential oil (200 *µ*g/mL) or eugenol (200 *µ*g/mL) and either stimulated or not stimulated with *T. cruzi* trypomastigotes (5 × 10^7^ parasites/mL). After 48 hours of incubation, the supernatant of cultures was collected and the analysis of nitrite quantification was carried out with Griess reagent. Briefly, in 96-well plates, 50 *µ*L of culture supernatant and 50 *µ*L of Griess reagent were added (25 *µ*L of sulfanilamide 1% in 2.5% H_3_PO_4_ solution and 25 *µ*L of N-(1-naphthyl)ethylenediamine 0.1% solution), followed by incubation in a dark environment for 10 minutes, and read at 570 nm on the spectrophotometer. The nitrite values were obtained from the standard curve of sodium nitrite (100–1.5 *µ*M) [24].

### 2.16. Statistical Analysis

The numerical results from at least two independent assays were expressed as mean ± standard deviation, and the IC_50_ and CC_50_ determination were performed with the GraphPad Prism 7.00 software package (GraphPad Software, San Diego, CA, USA). The Mann–Whitney test was used to analyze the results, and the difference at *p* < 0.05 was considered as significant.

## 3. Results

### 3.1. Chemical Composition of *S. aromaticum* Essential Oil

**Figure 1:**
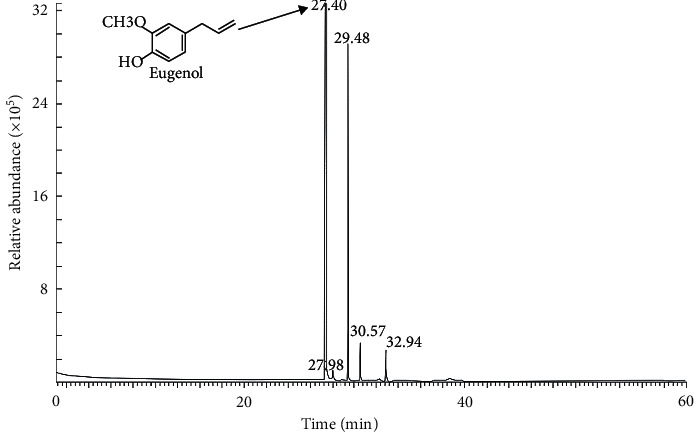
Chromatogram of *Syzygium aromaticum* essential oil.

The essential oil presented a yield of 3.8%, a density of 0.989 g/mL at 25°C, and a refractive index (ND 25) of 1.595. It was soluble in 90% (v/v) ethanol at a ratio of 1 : 2 and exhibited a transparent yellow color with a clear appearance in all samples. Chemical compounds in *S. aromaticum* essential oil evaluated by GC-MS are shown in Figure 1. Five major compounds were determined in the *S. aromaticum* essential oil and enumerated in order of elution and retention time. The major constituent was eugenol, representing 52.53% (Table 1).

### 3.2. Antioxidant Activity of *S. aromaticum* Essential Oil

**Figure 2:**
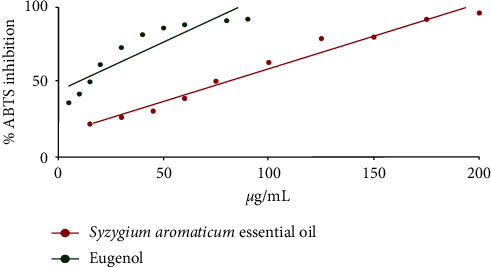
Inhibition of the ABTS radical by *Syzygium aromaticum* essential oil and eugenol.

*Syzygium aromaticum* essential oil and eugenol presented concentration-dependent antioxidant activity, as observed in the graph that relates *S. aromaticum* essential oil and eugenol concentration to the percentage of inhibition of the ABTS radical (Figure 2). The calculated EC_50_ was 78.98 *µ*g/mL for *S. aromaticum* essential oil and 12.66 *µ*g/mL for eugenol.

### 3.3. Antimicrobial Activity of *S. aromaticum* Essential Oil

**Figure 3:**
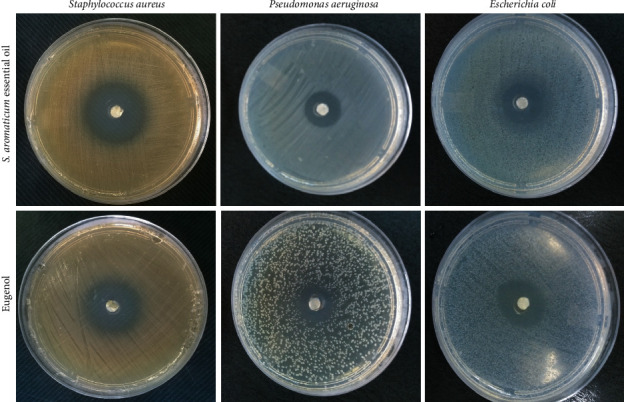
Inhibition zones of *Syzygium aromaticum* essential oil and eugenol on different bacterial cultures after 24 hours of treatment.

To assess antimicrobial activity, the disk diffusion test was performed. When checking the diameter of inhibition halo against Gram-positive (*S. aureus*) and Gram-negative (*E. coli* and *P. aeruginosa*) bacteria, the essential oil and eugenol showed a greater halo against *S. aureus* (Figure 3). The MIC assay revealed the significant antimicrobial activity of *S. aromaticum* essential oil, showing a similar effect against *E. coli* and *P. aeruginosa* and being more effective against *S. aureus* than eugenol (Table 2).

### 3.4. Antitrypanosomal Activity, Cytotoxicity, and Selective Index of *S. aromaticum* Essential Oil

**Figure 4:**
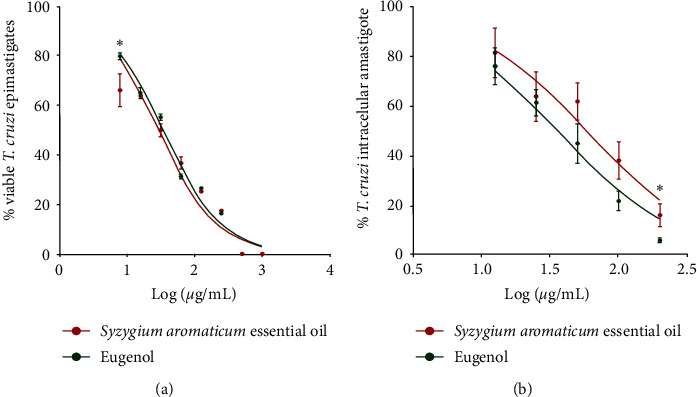
Dose-response curve of antitrypanosomal activity of *Syzygium aromaticum* essential oil and eugenol. Activity against epimastigotes (a) and intracellular amastigote of *Trypanosoma cruzi* (b) after 72 and 24 hours of treatment, respectively. Data represent mean ± standard deviation of the experiment realized in triplicate; ^*∗*^*p* < 0.05 when compared by Mann–Whitney test.

The inhibitory concentrations of *S. aromaticum* essential oil and eugenol against epimastigotes and intracellular amastigotes of *T. cruzi* are presented in Table 3. Both compounds presented concentration-dependent inhibitory activity, presenting differences in growth inhibition on epimastigote forms only at 7.8 *μ*g/mL (*p* = 0.0321) (Figure 4(a)). The similar inhibition activity of *S. aromaticum* essential oil and eugenol was evidenced by the absence of statistical differences between their IC_50_ values. In contrast, activity against intracellular amastigotes showed slightly larger differences in *S. aromaticum* essential oil and eugenol, with eugenol exhibiting greater activity than essential oil at the highest analyzed concentration (*p* = 0.0286) (Figure 4(b)). Compared to epimastigote activity, both compounds resulted in a reduction in intracellular amastigote inhibition, with *S. aromaticum* essential oil displaying a 2.2-fold increase in IC_50_ value against intracellular amastigotes compared to the IC_50_ against epimastigote forms.

Cytotoxicity assay revealed that *S. aromaticum* essential oil did not exhibit toxicity for BALB/c peritoneal macrophages even at the highest concentration analyzed (2,000 *μ*g/mL). Eugenol displayed greater cytotoxicity than *S. aromaticum* essential oil, directly influencing its selectivity index. Thus, although eugenol exhibited better activity against intracellular amastigotes, *S. aromaticum* essential oil exhibited a higher SI value (Table 3).

**Figure 5:**
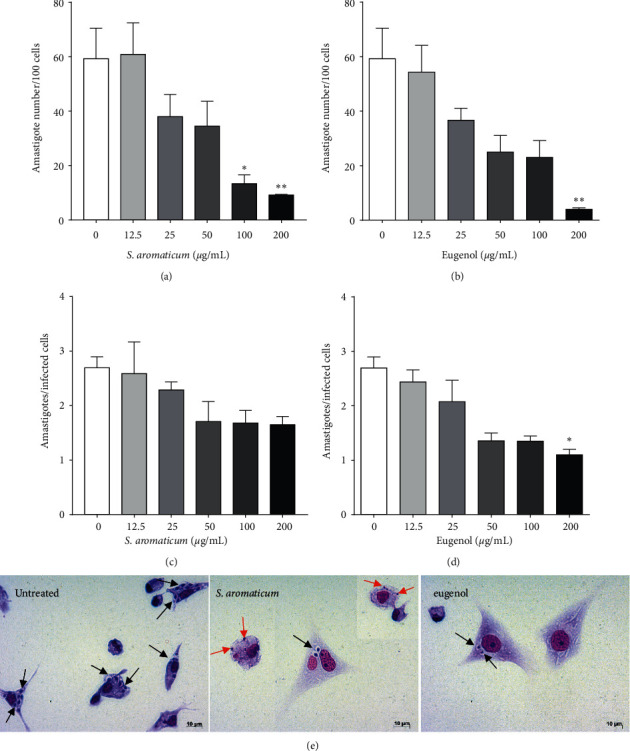
BALB/c peritoneal macrophages infected with *Trypanosoma cruzi* and treated with *Syzygium aromaticum* essential oil or eugenol for 24 hours. Parameters of infection (a-d) and light microscopy (e). Intracellular amastigotes (black arrows) and remains of amastigotes (red arrows) inside macrophages. The images and data (mean ± standard deviation) represent two independent experiments performed in quadruplicate. ^*∗*^*p* < 0.05 and ^*∗*^^*∗*^*p* < 0.01 when compared with the untreated control group by the Mann–Whitney test. Giemsa, 40x objective.

The parameters of infection analysis (Figure 5) showed that *S. aromaticum* essential oil treatment significantly reduced the number of amastigotes per 100 cells at 200 *μ*g/mL (*p* = 0.0068) and 100 *μ*g/mL (*p* = 0.0460) (Figure 5(a)), while eugenol reduced the number of amastigotes per 100 cells (*p* = 0.0095, Figure 5(b)) and the mean number of amastigotes per infected cells (*p* = 0.0112, Figure 5(d)) at 200 *μ*g/mL. The reductions in intracellular amastigotes of *T. cruzi* after treatment with *S. aromaticum* essential oil and eugenol can be seen in Figure 5(e).

### 3.5. *S. aromaticum* Essential Oil Induces Nitrite Levels Reduction in *T. cruzi*-Infected Peritoneal Macrophages

**Figure 6:**
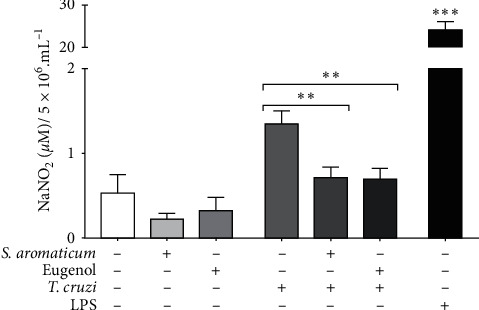
Nitrite quantification in the supernatant of the BALB/c peritoneal macrophage treated with *Syzygium aromaticum* essential oil (200 g/mL) or eugenol (200 g/mL) and stimulated or not with *Trypanosoma cruzi*. Data represent mean ± standard deviation of experiment realized in sextuplicate; ^*∗*^^*∗*^*p* < 0.01, ^*∗*^^*∗*^^*∗*^*p* < 0.001 when compared with untreated and unstimulated macrophages or, between brackets, with the Mann–Whitney test.

Nitrite levels were measured in the supernatant of BALB/c peritoneal macrophages and showed a decrease in cells treated with *S. aromaticum* essential oil (0.22 ± 0.067 *μ*M NaNO_2_) and eugenol (0.32 ± 0.155 *μ*M NaNO_2_), with reductions of 57.69% and 38.46%, respectively, when compared to the untreated cells (0.52 ± 0.224 *μ*M NaNO_2_), although these reductions were not statistically significant. However, a significant reduction in the nitrite levels of cells stimulated with *T. cruzi* and treated with *S. aromaticum* essential oil (0.71 ± 0.123 *μ*M NaNO_2_, *p* = 0.002) and eugenol (0.69 ± 0.126 *μ*M NaNO_2_, *p* = 0.003) was observed, with reductions of 47.01% and 48.05% respectively, when compared to stimulated and untreated cells (1.34 ± 0.152 *μ*M NaNO_2_) (Figure 6).

## 4. Discussion

This research studied the chemical composition and antimicrobial, antioxidant, and antitrypanosomal activity of *S. aromaticum* essential oil, describing for the first time its activity against intracellular amastigotes of *T. cruzi* and its inhibitory effect on *T. cruzi*-stimulated peritoneal macrophages. The physical-chemical parameters (color, appearance, solubility, density, and refractive index) were evaluated to ensure the integrity and quality of the essential oil, as environmental factors (light, heat, air, and humidity) can influence the change in the chemotype of plant species. The physical characteristics of *S. aromaticum* essential oil were similar to the patterns described in previous studies [25].

Numerous studies have identified and quantified similar chemical compounds in the essential oils of *S. aromaticum*, revealing that this species has a similar chemotype to eugenol-rich chemotype. Eugenol was identified as a major compound (90.3%) in an essential oil extracted from the south of Brazil, in addition to *β*-caryophyllene (4.83%) and eugenol acetate (1.87%) [26], while another study, also on essential oil of cloves from the south of Brazil, observed the presence of eugenol (56.06%) and caryophyllene (39.63%) in greater quantities [27], very similar to the amount found in the present study. *S. aromaticum* essential oils obtained in China [28] and Italy [29] also had eugenol as their major compound, with 90.84% and 77.9%, respectively. The amount of eugenol contained in the essential oil and the difference between the compounds can be directly related to the different geographic areas where the plant has grown up, which can be influenced or changed by biotic and abiotic factors such as seasonality, stage of development, age of the plant, and climatic conditions [30]. In addition, the extraction method used to obtain the oil can also affect its chemical composition, as distillation and storage conditions are capable of influencing the content of its volatile metabolites [31]. Differences in chemical composition can be directly related to pharmacological properties, as noted in the antimicrobial activity tests in the present study.

*S. aromaticum* essential oil was active against standard strains of Gram-negative and Gram-positive bacteria, showing inhibition halo ranges between 14 and 25 mm by the disk diffusion test. These results agree with data from literature, in which a clove essential oil of more than 90% eugenol was more efficient against Gram-negative bacteria but showed smaller halos than those in our research for Gram-positive bacteria [26]. Another eugenol-rich clove essential oil presented inhibition zones of 28.3 and 28.1 mm for *S. aureus* and *E. coli*, respectively, indicating the susceptibility of the bacteria to the essential oil [27]. The study by Cimanga et al. [32] used inhibition halo size (IHS) to classify antimicrobial activity as follows: IHS≥15 mm strong inhibition; 10≤IHS <15 moderate inhibition; and IHS <10 inactive. When the results of the present study are compared with literature, a pattern can be observed, with *S. aromaticum* essential oil being considered as strong inhibitor for *S. aureus* and *E. coli* and a moderate inhibitor for *P. aeruginosa*.

The inhibition halos induced by *S. aromaticum* essential oil were higher than eugenol standard. It has been reported that essential oil possesses antimicrobial activity due to the presence of eugenol, which is described as the most important component of cloves, but such activity has also been found to be due to other phenolic compounds [33, 34]. Our results make it evident that the biocomplex is responsible for such activity, reinforcing the results of MIC determination.

The MIC of *S. aromaticum* essential oil displayed values between 50 and 200 *μ*g/mL. Holetz et al. [35] classified samples with MIC values below 100 *μ*g/mL as presenting good antibacterial activity, from 100 to 500 *μ*g/mL as moderate activity, and from 500 to 1000 *μ*g/mL as weak activity and those above 1000 *μ*g/mL as inactive. Based on this, the essential oil analyzed in this study exhibited good inhibitory activity against the *S. aureus* strain and moderate activity against *E. coli* and *P. aeruginosa*. Eugenol demonstrated moderate activity against all the strains in the present study. When our data was compared with previous work, it was observed that a Brazilian eugenol-rich clove essential oil had MIC value of 60 *μ*g/mL for the *E.coli* and *S. aureus* strain [36], a result close to that found in our study for the Gram-positive strain, while a clove essential oil with more than 90% eugenol resulted in MIC of 1,318 *μ*g/mL for *E.coli* [29]. Eugenol was analyzed against an *E.coli* strain and exhibited 3,284 *μ*g/mL [37] and 1,600 *μ*g/mL MIC values [38]. Different results from those found in the present study may be related to the type of bacterial culture used. Eugenol was evaluated against standard strains and clinical isolates of *S. aureus* presenting MIC value of 256 *μ*g/mL for the standard strain, while the other 25 strains had MIC values ranging from 128 to 512 *μ*g/mL [39].

Selles et al. verified eugenol as the major compound of clove and found that EC_50_ value of 4.82 ± 0.06×10^−2^ *µ*g/mL for *S. aromaticum* essential oil eliminated the DPPH radical, less than the value found in our research, while the MIC against bacteria obtained in this study ranged from 1.36 to 2.72 mg/mL, values lower than those found in our study [40]. Kaur et al. observed the presence of twenty-one compounds with eugenol as the main compound, also verifying the antioxidant activity of clove essential oil by DPPH with EC_50_ in the range of 10.87 to 31.63 *μ*g/mL [41].

Eugenol is a chemotype found in several plant products and is one of the main constituents of clove essential oil. It is used in both food and cosmetics industries as a flavoring agent, and it is an excellent antimicrobial, having activity against fungi and bacteria [42]. Eugenol has free hydroxyls in its structure that may be responsible for the antimicrobial activity verified in this research, since the antimicrobial activity of nitric oxide (NO) is conferred by its free hydroxyl groups. It was deduced that the hydroxyl group in eugenol links to proteins, preventing enzymatic action. The cell membrane suffered ruptures in the presence of the essential oil because it is rich in lipophilic compounds. This damage directly affects the maintenance of cellular pH and the balance of the inorganic ions. The main factors responsible for this damage are monoterpenes and sesquiterpenes, which produce different effects in different microorganisms [43].

Eugenol and *S. aromaticum* essential oil demonstrated great capacity for the elimination of ABTS radical, agreeing with the data reported on the antioxidant activity of clove essential oils with a chemotype rich in eugenol [44]. The same result was observed in a study of the antioxidant activity of the essential oil from various parts of the plant *S. aromaticum,* which demonstrated its ability to reduce the ABTS radical [45].

Medicinal plants have shown therapeutic components in their composition with potential candidates for new drugs [46]. Among these components, essential oils and secondary plant metabolites are considered as alternatives or additions to antiparasitic therapies [47]. The trypanocidal activity of several essential oils is described in literature [48, 49]. Due to the challenges in the development of new therapeutic agents for Chagas disease, we reported the effect of *S. aromaticum* essential oil and its major component eugenol on the growth of *T. cruzi* epimastigotes and intracellular amastigotes.

In the present study, the effects obtained when *T. cruzi* was treated with clove essential oil showed that epimastigotes are more susceptible to treatment than intracellular amastigotes. The activity of *S. aromaticum* essential oil may be in part attributed to the presence of eugenol, which makes up over half its composition. Thus, in an attempt to identify the compound responsible for the activity observed in the essential oil, the eugenol was analyzed. Eugenol demonstrated antitrypanosomal activity when incubated with *T. cruzi*; however, due to its cytotoxicity on macrophages, eugenol becomes less selective than *S. aromaticum* essential oil.

There are reports that *S. aromaticum* exhibited activity against trypomastigote forms of *Trypanosoma brucei brucei*, with IC_50_ values of 10.4 *μ*g/mL. However, it also exhibited cytotoxicity on macrophages (CC_50_ 22.4 *μ*g/mL) and low SI (2.2) [50], unlike what was observed in the present study. A study analyzed *S. aromaticum* essential oil against *T. cruzi* (Y strain), obtaining IC_50_/24 h values of 99.5 *μ*g/mL for epimastigotes and 57.5 *μ*g/mL for bloodstream trypomastigotes. Eugenol was also evaluated, presenting IC_50_/24h values of 246 *μ*g/mL for epimastigotes and 76 *μ*g/mL for trypomastigotes [51].

A similarity was observed between the inhibitory activities of *S. aromaticum* essential oil and eugenol against *T.* cruzi epimastigote, and so activity against intracellular amastigotes was also analyzed. To our knowledge, this is the first description of *S. aromaticum* essential oil activity against *T. cruzi* intracellular amastigote forms. As there is a lack of data about *S. aromaticum* essential oil activity against *T. cruzi* intracellular amastigotes in literature, we found this activity similar to that observed against intracellular amastigotes of *Leishmania donovani* (15.24 *μ*g/mL) [52], a protozoa that belongs to the same Trypanosomatidae family of *T. cruzi*. Eugenol displayed better activity against the intracellular amastigotes than *S. aromaticum* essential oil. Parameters of infection analysis corroborated the intracellular amastigote results, showing that eugenol was able to decrease the number of amastigotes per 100 cells and the mean amastigotes per infected cells, while *S. aromaticum* essential oil only decreased amastigote numbers per 100 cells, although in a concentration inferior to that presented by eugenol.

The cell death mechanism of intracellular amastigotes may be an event associated with the activation of macrophage microbicidal mechanisms, particularly the increase in the production of NO levels [53], which can be indirectly measured by the quantification of nitrite in the supernatant of BALB/c peritoneal macrophages. Therefore, in an attempt to better understand activity against intracellular amastigote forms, we carried out an analysis of the nitrite quantification of *T. cruzi*-stimulated peritoneal macrophages treated with *S. aromaticum* essential oil or eugenol. A significant decrease in nitrite levels was observed in cells stimulated with *T. cruzi* and treated with essential oil or eugenol. Eugenol notably modulates NO production, inducing suppression of NO production and iNOS protein expression in carbon tetrachloride-induced liver injury in rats [54] and in nicotine-induced murine peritoneal macrophages [55]. Eugenolol and glyceryl-isoeugenol, two derivatives of eugenol, suppressed LPS-induced iNOS expression by downregulating NF-kB and AP-1 through the inhibition of the MAPKs and Akt/IkB-alpha signaling pathways [56]. The inhibition of NO production observed in macrophages treated with *S. aromaticum* essential oil is probably due to the known NO suppression by eugenol. Therefore, there are probably other possible mechanisms involved in the trypanocidal activity of *S. aromaticum* essential oil against intracellular amastigotes, which are not involved in nitric oxide. Further studies should elucidate these mechanisms.

## 5. Conclusions

Analysis of the chemical composition of *S. aromaticum* essential oil identified eugenol as its major compound. *S. aromaticum* essential oil and eugenol inhibit the growth of Gram-positive bacteria *S. aureus* and have antioxidant potential. Inhibitory activity against the epimastigotes and intracellular amastigotes of *T. cruzi*, associated with low cytotoxicity, demonstrated the selectivity of *S. aromaticum* essential oil against the parasite. In addition, the NO inhibition observed in *T. cruzi*-stimulated macrophages treated with *S. aromaticum* essential oil showed that there are probably other possible mechanisms involved in their trypanocidal activity, which are not related to NO. *S. aromaticum* essential oil trypanocidal activity provides optimism about its use in further research seeking new therapeutic alternatives to trypanosomiasis.

## Figures and Tables

**Table 1:** Chemical composition of *Syzygium aromaticum* essential oil.

Peak	Compounds	RT (min)^1^	Kovats index	PA (%)^2^
1	Eugenol	27.40	1389	52.53
2	Copaene	27.98	1368	2.05
3	Caryophyllene	29.48	1538	37.25
4	Humulene	30.57	1498	4.11
5	Eugenyl acetate	32.94	1515	4.05

^1^Retention time. ^2^Peak area percentage in relation to peak total area.

**Table 2:** Inhibitory zone diameters and minimum inhibitory concentration of *Syzygium aromaticum* essential oil and eugenol on different bacterial cultures after 24 hours of treatment.

Antimicrobial assay	Compounds	Bacteria strain		
		*E. coli*	*S. aureus*	*P. aeruginosa*
Inhibition zones (mm)	*S. aromaticum*	22.67 ± 0.577^a^	25.00 ± 1.000^a^	14.00 ± 1.000^a^
	Eugenol	20.33 ± 0.577^a^	22.33 ± 0.577^b^	11.00 ± 1.000^b^
	Gentamycin	13.33 ± 1.155^b^	20.33 ± 0.577^b^	16.67 ± 0.577^c^
	Ampicillin	13.67 ± 0.577^b^	—	8.33 ± 1.155^d^

MIC (*μ*g/mL)	*S. aromaticum*	100.0 ± 0.00^a^	50.0 ± 0.00^a^	200.0 ± 0.00^a^
	Eugenol	100.0 ± 0.00^a^	250.0 ± 0.00^b^	200.0 ± 0.00^a^
	Gentamycin	16.00 ± 0.00^b^	8.00 ± 0.00^c^	–
	Polymyxin B	—	—	16.00 ± 0.00^b^

MIC: minimum inhibitory concentration. Data represent mean ± standard deviation of experiment carried out in triplicate. Different letters in the same column in each antimicrobial assay mean statistical difference between groups.

**Table 3:** Antitrypanosomal activity, BALB/c peritoneal macrophage cytotoxicity, and selectivity index of *Syzygium aromaticum* essential oil.

Compound	*Trypanosoma cruzi* IC_50_ (*μ*g/mL)		Peritoneal macrophage CC_50_ (*μ*g/mL)	SI
	Epimastigote	Intracellular amastigote		
*S. aromaticum* essential oil	28.68 ± 1.073^a^	64.51 ± 1.658^a^	>1000^a^	>15.5^a^
Eugenol	31.97 ± 1.061^a^	45.73 ± 1.252^b^	292.7 ± 1.229^b^	6.4^b^
Benznidazole	1.950 ± 1.066^b^	0.4726 ± 1.163^c^	187.2 ± 1.125^c^	396.1^c^

IC_50_: inhibitory concentration for 50% of parasites; CC_50_: cytotoxic concentration for 50% of cells; SI: selectivity index, obtained from ratio CC_50_/IC_50_ intracellular amastigote. Data represent mean ± standard deviation of at least two independent experiments carried out in triplicate. Different letters in the same column mean statistical difference between groups.

## Data Availability

The data used to support the findings of this study are included within the article.
